# Positive sputum fungal culture, fungal sensitisation, and airway microbial diversity in asthmatic children

**DOI:** 10.1093/mmy/myaf005

**Published:** 2025-01-24

**Authors:** Paul J Seear, Kathryn G Welsh, Jack Satchwell, Deepa Patel, Catherine H Pashley, Andrew J Wardlaw, Erol A Gaillard

**Affiliations:** Department of Respiratory Sciences, University of Leicester, Leicester, UK; Department of Respiratory Sciences, University of Leicester, Leicester, UK; Department of Respiratory Sciences, University of Leicester, Leicester, UK; Department of Respiratory Sciences, College of Life Sciences, NIHR Biomedical Research Centre (Respiratory Theme), University of Leicester, Leicester, UK; Department of Paediatric Respiratory Medicine, University Hospitals of Leicester NHS Trust, Leicester, UK; Department of Respiratory Sciences, University of Leicester, Leicester, UK; Department of Respiratory Sciences, University of Leicester, Leicester, UK; Department of Respiratory Sciences, College of Life Sciences, NIHR Biomedical Research Centre (Respiratory Theme), University of Leicester, Leicester, UK; Department of Paediatric Respiratory Medicine, University Hospitals of Leicester NHS Trust, Leicester, UK

**Keywords:** paediatric, lung, mycobiome, microbiome, *Aspergillus fumigatus*

## Abstract

Sensitisation to thermotolerant fungi such as *Aspergillus fumigatus* and *Candida albicans*, which can colonise the airways, is associated with poor lung function in children with asthma. Dysbiosis of bacteria and fungi in the airway microbiome has been reported between health and asthma but has yet to be characterised for fungal-sensitised asthmatic children. We investigated if microbial diversity of the airways is altered in fungal-sensitised school-age asthmatic children. Sputum samples from children with asthma who were fungal sensitised (*n* = 22) and non-fungal sensitised (*n* = 17) along with children without asthma (*n* = 15), aged 5–16 years were profiled by traditional microbiological culture, modified fungal culture, bacterial 16S, and fungal ITS2 next-generation sequencing. Microbiota were compared between groups using alpha/beta diversity and differential abundance analysis. Bacterial alpha diversity was significantly lower in asthma compared to disease controls and in stable compared to acute asthma. Fungal alpha and beta diversity did not change between asthma states and disease controls, but alpha diversity was significantly lower in asthma samples from patients with positive *A. fumigatus* culture. Children sensitised to fungi had similar microbial diversity compared to non-sensitised children. However, in children not sensitised to fungi, those with a positive airway fungal culture had significantly lower fungal alpha diversity and bacterial beta differences compared to children with negative fungal culture. Fungal sensitisation did not alter bacterial or fungal microbiota in the airways of asthmatic children. However, positive airway fungal culture was associated with significant changes in microbial diversity, particularly in non-fungal sensitised children with asthma.

## Introduction

Asthma is a chronic inflammatory airways disorder characterised by variable airflow obstruction and bronchial hyper-responsiveness. Worldwide, asthma affects between 12 and 14% of children aged 6–14 years,^1^ and recent European data suggest that 2.1–10% of the paediatric asthma population have severe asthma.^2^

High rates of children and adults with severe asthma are IgE sensitised to fungi,^3–5^ and both fungal sensitisation^6^ and the presence of fungi in the sputum of asthmatics^7^ are associated with worse lung function^8–10^ and more severe disease. In particular, IgE sensitisation along with colonisation by thermotolerant fungi that are able to colonise the airways such as *Aspergillus fumigatus* and *Candida* species can lead to a number of conditions such as lung fibrosis, bronchiectasis, and fixed airflow obstruction that have been grouped under the term allergic fungal airways disease (AFAD^11,12^). Over time, airway fungal colonisation in association with IgE sensitisation appears to cause damage to the lung including the development of bronchiectasis, lung fibrosis, and fixed airflow obstruction.^5,6,13^ The mechanisms leading to the more rapid decline in lung function in patients sensitised to and airway colonised with fungi have not been fully established, but fungi have been shown to promote production of T-helper 2 cell-associated cytokines and mucin production.^14^ In addition, cohort studies investigating the airway microbiome reported an association between microbial dysbiosis and asthma with strong evidence of a complex polymicrobial community within the airways in both healthy lungs and in chronic asthma.^5,15–19^

However, few studies reported on the association between the airway microbiome and the lung fungal mycobiota in asthma. This interaction may play an important role in the pathophysiology of asthma patients with fungal sensitisation and airway colonisation. Recently, Huang et al.^17,18^ reported a lower fungal diversity in adult patients with untreated asthma relative to controls, and Rick et al.^19^ described the airway mycobiota in adult asthmatics with and without fungal sensitisation. One study in children compared mycobiota and microbiota between children with severe asthma and healthy controls in a small case series^20^ reporting specific differences in both the lung microbiome and mycobiome between the two groups but no increase in bacterial diversity. To our knowledge, this is the first study investigating the impact of fungal IgE sensitisation and fungal airway colonisation on the airway microbiome in children with asthma.

## Methods

### Participant recruitment and sputum collection

Children aged 5–16 years with a confirmed diagnosis of asthma based on a combination of clinical symptoms, spirometry, and bronchodilator reversibility were recruited from University Hospitals of Leicester (UHL) NHS Trust, Leicester, UK between 2013 and 2016. Paediatric asthma participants were recruited from the UHL emergency department and stable asthmatics from the UHL severe paediatric asthma clinic. Institutional review board approval was obtained from the NRES Committee West Midlands – Edgbaston. Disease-control children aged between 5 and16 years, with no history of pre-existing respiratory conditions, including wheeze and atopy, were recruited from paediatric diabetes outpatient clinics as part of our asthma biomarker study (FAST study REC 12/WM/0413). The term atopy refers to the predisposition to developing allergic diseases such as allergic rhinitis, asthma, and eczema, and therefore participants with a history of this were excluded. All participants underwent standard clinical investigations, spirometry, and fungal sensitisation were determined against a panel of five fungi, and routine microbiology was supplemented by a fungal culture protocol optimised towards the detection of filamentous fungi from sputum samples. Children with chronic asthma and control children attended a single visit to the paediatric respiratory laboratory for spirometry and allergy testing and sputum induction with hypertonic saline. Children attending the emergency department with acute asthma were invited to produce a sputum sample; either spontaneously following bronchodilation or after 0.9% saline nebulisation as previously described.^21^ Full details on participant recruitment, allergy testing, sputum collection, and processing along with fungal culture are provided in Welsh et al.^5^ Briefly, fungal and aeroallergen sensitisation were tested with either skin prick test (SPT) (Soluprick, ALK-Abello, Hørsholm, Denmark) or sandwich immunoassay. The SPT was deemed positive where the weal size was ≥3 mm greater than control.^22^ Serum total IgE and specific IgEs were quantified using the ImmunoCap250 system (Pharmacia, Milton Keynes, UK).

### Patient groups

Paediatric asthma patients were categorised into sensitised and non-sensitised groups based on total IgE and fungal-specific IgE. The cutoff for total serum IgE in asthma has not been strictly defined; however, the UK has used a cutoff of >180 IU/ml.^23^ At UHL, a total IgE level >75 kU/l is classed as high in combination with a positive fungal-specific IgE greater than 0.35 kU/l. Participants with asthma were classified into a disease severity category based on the Global Initiative for Asthma (GINA) treatment pathway.^24^ The categorisation ranges from steps 1 to 5 based on a combination of clinical symptoms and pharmacological treatment.

### Statistical analysis of demographic and clinical characteristics

R version 4.2.1 was used to perform all statistical analyses of subject demographic and clinical data. Continuous parametric data were analysed using an independent two-tailed *t*-test or two-way anova, whilst non-parametric data were analysed using Mann–Whitney *U* test or Kruskal–Wallis test for two groups and three groups, respectively. Categorical data were analysed using *χ*^2^ test or Fisher’s exact test. A *P*-value of less than .05 was considered significant.

### Library preparation and sequencing

Extraction of DNA from 100 µl of homogenised sputum samples was performed using the DNeasy® Plant Mini Kit (Qiagen) modified with a 2 min bead beating step.^19,25^ A dual index nested PCR approach was used to amplify the internal transcribed spacer region 2 (ITS2) of fungi and the V4 region of bacterial 16S ribosomal RNA using barcoded TruGrade (Integrated DNA Technologies Inc., USA) ITS3/ITS4 primers^26^ and F515/R806 primers,^27^ respectively. For full details, see Supplementary Method S1. Bacterial 16S amplicons and fungal ITS2 amplicons were submitted to the Centre for Genomic Research, University of Liverpool for 2 × 250 bp and 2 × 300 bp paired-end sequencing, respectively, on the Illumina MiSeq platform.

### Bioinformatic and statistical analysis

Sequence pre-processing was performed as described in Rick et al.^19^ Further processing using DADA2^28^ in QIIME2-2022.2^29^ corrected sequencing errors, removed chimerics and singletons, and dereplicated sequences to generate amplicon sequence variants (ASVs). Sequencing contaminants were identified and removed using the decontam package (default parameters) in R.^30^ Samples with less than 1000 ASVs were removed. All further analyses were performed in QIIME2-2022.2. Sequence data were deposited in the European Nucleotide Archive; study accession numbers PRJEB60938 (ITS2) and PRJEB60939 (16S). In order to identify taxa that were differentially abundant between sample groups, analysis of composition of microbiomes (ANCOM) that accounts for the compositional nature of high-throughput sequencing (HTS) data was performed using unrarefied ASV tables.^31^ Full details on bioinformatic and statistical analyses are provided in the Supplementary Method S1.

## Results

### Participants and samples

DNA was extracted from the sputum of 15 controls without asthma and 41 individuals with asthma, of which 22 were IgE sensitised to fungi, with 17 not fungal sensitised (no sensitisation data available in two individuals, see Table 1). Compared to non-fungal-sensitised and disease controls, fungal-sensitised asthma individuals had the highest serum total IgE (*P* = .001), the highest proportion of children atopic to non-fungal aeroallergens (*P* = .0002), and all were atopic to both fungal allergens (*Candida, Penicillium, Aspergillus fumigatus, Cladosporium*, and *Alternaria*) and non-fungal allergens such as cat, dog, grass, and house dust mites (*P* < .0001). Post-bronchodilator FEV1 and FEV1/FVC ratios were significantly higher in the controls (*P* = .009). Children with asthma sensitised to fungi and disease controls were older compared to the non-sensitised group. Between the three groups, there were no significant differences in positive culture of fungi from sputum, although culture rates were low overall.

**Table 1.** Demographics and clinical characteristics.

Variable	Participants with asthma (*n* = 41)					*n*	Control, no asthma (*n* = 15)	*n*	*P-*value
Male, *n* (%)^a^	24 (59%)					41	5 (33%)	15	.17
Age (years), mean (range)^c^	11 (5–16)					41	12 (6–16)	15	.04
GINA treatment									
GINA 1–3, *n* (%)	21 (51%)					41	–	–	–
GINA 4–5, *n* (%)	20 (49%)					41	–	–	–
PB FEV_1_*z* score, mean (SD)^e^	−0.66 (1.29)					37	−0.29 (1.1)	12	.47
PB FEV_1_/FVC, mean (SD)^e^	0.81 (0.09)					37	0.9 (0.06)	12	.004
Eosinophil count, median (IQR), median (interquartile range)^e^	0.51 (0.27–0.78)					35	0.17 (0.1–0.31)	11	.03
Serum total IgE (KU/l)^e^, median (interquartile range) ^e^	534 (111–1340)					35	57 (23–134)	9	.003
Samples analysed by high-throughput sequencing^g^									
16S, *n* (%)^b^	36 (88%)					41	15 (100%)	15	.665
ITS, *n* (%)^a^	28 (68%)					41	8 (53%)	15	.472
	Fungal sensitisation (*n* = 39)^a^								
	Fungal sensitised (*n* = 22)^a^	*n*	Non-fungal sensitised (*n* = 17)^a^	*n*	*P-*value	Control, no asthma (*n* = 15)^a^		*n*	*P-*value
Male, *n* (%)^a^	15 (68%)	22	8 (47%)	17	.317	5 (33%)		15	.102
Age (years)^c,d^, mean (range)	12 (6–16)	22	9 (5–15)	17	.02	12 (6–16)		15	.007
GINA treatment									
GINA 1–3, *n* (%)^a^	9 (41%)	22	11 (65%)	17	.25	–		–	
GINA 4–5, *n* (%)^a^	13 (59%)	22	6 (35%)	17					
PB FEV_1_*z* score^e,f^, mean (SD)	−0.96 (1.34)	20	−0.26 (1.2)	15	.12	-0.29 (1.1)		12	.215
PB FEV_1_/FVC^e,f^, mean (SD)	0.8 (0.1)	20	0.84 (0.08)	15	.24	0.9 (0.06)		12	.0085
Serum total IgE (KU/l)^e,f^, median (interquartile range)	766 (374–1556)	21	164 (70.8–606)	12	.04	57 (23–134)		9	.001
Daily ICS dose (µg)^e^, median (interquartile range)	800 (400–1000)	22	400 (400–1000)	17	.47	–		–	–
Mould in home *n* (%)^a^	10 (50%)	20	7 (50%)	14	1	10 (67%)		15	.558
Sputum culture positive									
Yeast, *n* (%)^a^	7 (32%)	22	6 (35%)	17	1	4 (27%)		15	.933
*A. fumigatus, n* (%)^a^	9 (41%)	22	3 (18%)	17	.226	2 (13%)		15	.145
Other filamentous fungi, *n* (%)^b^	1 (5%)	22	1 (6%)	17	1	1 (7%)		15	1
Atopy^h^									
None, *n* (%)^b^	0	22	6 (35%)	17	<.0001	10 (67%)		15	<.0001
Non-fungal allergen only, *n* (%)^c,d^	21 (95%)	22	11 (65%)	17	.03	5 (33%)		15	.0002
Fungal allergen only	0	22	0	17		0		15	
Both categories of allergen, *n* (%)	22 (100%)	22	0	17		0		15	
Fungal sensitisation									
*A. fumigatus, n* (%)^b^	13 (59%)	22	0	17	<.001	0		15	<.001
Filamentous fungi, *n* (%)^b^	20 (90%)	22	0	17	<.0001	0		15	<.0001
Thermotolerant filamentous fungi, *n* (%)^b^	15 (68%)	22	0	17	<.0001	0		15	<.0001
Thermotolerant fungi, *n* (%)^b^	18 (82%)	22	0	17	<.0001	0		15	<.0001

Notes: Data analysed using ^a^*χ*^2^ test/^b^Fisher’s exact test (categorical), ^c^independent two-tailed *t*-test/^d^two-way anova (parametric), ^e^Mann–Whitney *U* test/^f^Kruskal–Wallis test (non-parametric). ^g^Samples >1000 ASVs. ^h^Skin prick test.

PB, post-bronchodilator; FEV1, forced expiratory volume in the first second; FVC, forced vital capacity; IgE, immunoglobulin E.

### Fungal ITS2 and bacterial 16S amplicons

HTS amplicons were obtained from most individuals with 51 bacterial 16S and 34 fungal ITS2 samples successfully sequenced following the removal of samples with less than 1000 processed reads (ASVs). The average number of ASVs per sample after quality control and removal of contaminants was 112 757 (range 1804–376011) for 16S and 95 549 (range 8644–301003) for ITS2. From these ASVs, taxonomic classification identified 268 bacterial taxa and 134 fungal taxa at the genus and species level, respectively. For the full list of alpha/beta diversity and ANCOM analysis results, see Supplementary Table 2.

### Relationship between microbial communities and clinical variables

To investigate if bacterial or fungal lung microbiota were associated with clinical characteristics, analyses were performed with and without disease controls, unless asthma specific.

#### Bacterial microbiota

**Figure 1.**
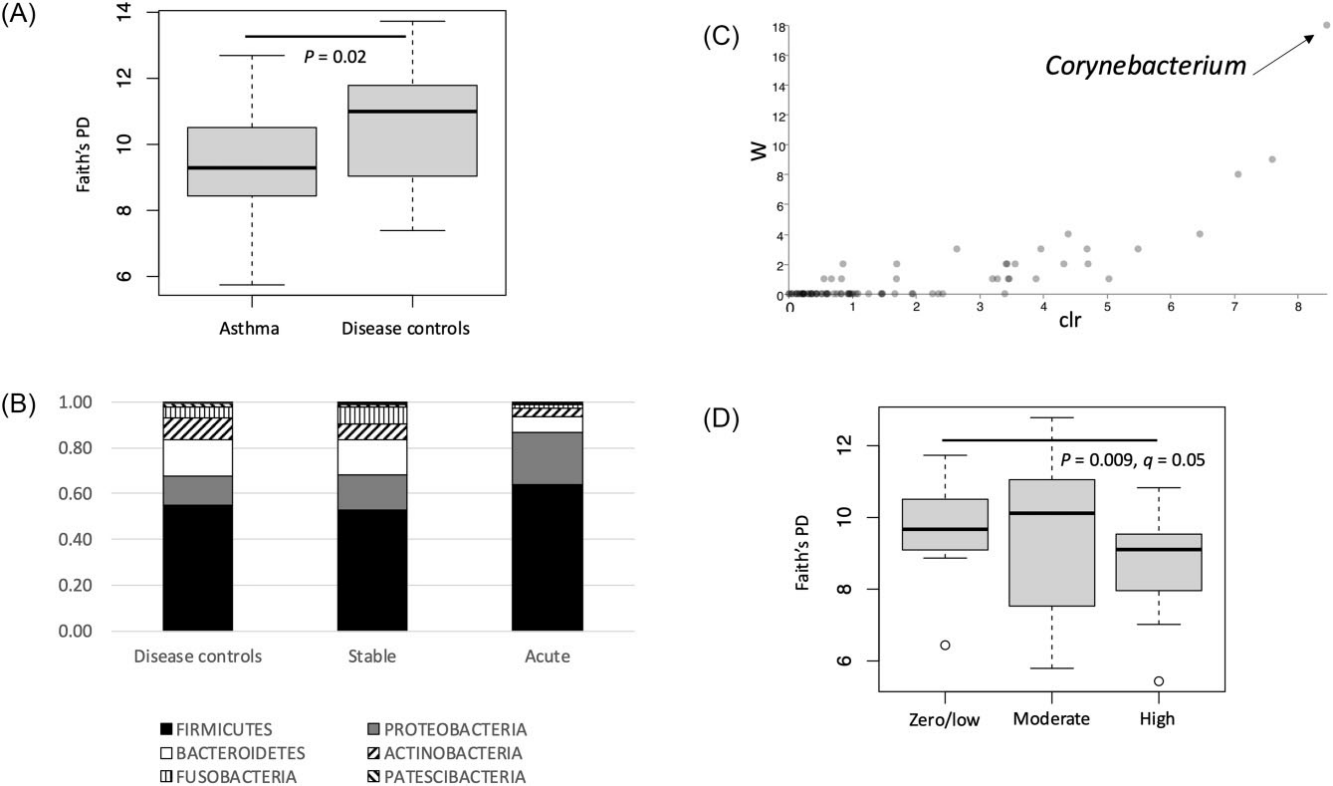
Relationship between airway bacterial microbiota and clinical variables. (A) Boxplot of Faith’s phylogenetic diversity indices showing a significant difference (*P* = .02) in bacterial alpha diversity between asthma (*n* = 34) and disease controls (*n* = 15). (B) Stacked plot showing relative abundances of bacterial phyla in disease controls compared to stable and acute asthma. (C) Volcano plot from the analysis of composition of microbiomes (ANCOM) test comparing asthma states and disease controls. The *y*-axis (*W* value) represents the number of times the null hypothesis (no change in abundance between groups) has been rejected, whilst the clr (centre log-ratio transformed) value on the *x*-axis represents the mean difference in abundance of a certain taxon between sample types. (D) Boxplot of Faith’s phylogenetic diversity indices showing a significant difference (*P* = .009, *q* = 0.05) in bacterial alpha diversity between zero/low (0–200 mg/day) and high doses (1000–2000 mg/day) of inhaled corticosteroids.

Figure 1A represents a box plot of differences in bacterial alpha diversity between asthma and disease control groups. The alpha diversity in the disease control cohort was significantly higher than in asthma (*P* = .02). Significant differences in alpha diversity were also noted between stable and acute asthma (*P* = .01; *q* = 0.03), with lower diversity in acute asthma. Figure 1B represents differences in bacterial taxa composition between disease control, stable asthma, and acute asthma. Acute asthma had the highest relative abundances of Firmicutes (acute 64%, stable 53%, disease control 55%) and Proteobacteria (acute 23%, stable 15%, disease control 13%). Acute asthma had the lowest Bacteroidetes (acute 7%, stable 16%, disease control 16%) and Actinobacteria (acute 4%, stable 7%, disease control 10%). Stable asthma had the highest relative abundance of Fusobacteria (acute 2%, stable 7%, disease control 5%).

ANCOM analysis is represented in Figure 1C. This figure highlights *Corynebacterium* as differentially abundant between asthma states and disease control (highest relative abundance in control and stable, lowest in acute). ANCOM also showed *Pseudomonas* was differentially abundant between yeast culture positive and negative in asthma only (highest in yeast culture positive). Figure 1D demonstrates bacterial alpha diversity between differing doses of inhaled corticosteroids. Participants with a high dose of ICS (*P* = .009, *q* = 0.05; Fig. 1D) and asthmatics in GINA treatment groups 4–5 (*P* = .005, *q* = 0.01) both had significantly lower alpha diversity than disease controls.

#### Fungal mycobiota

**Figure 2.**
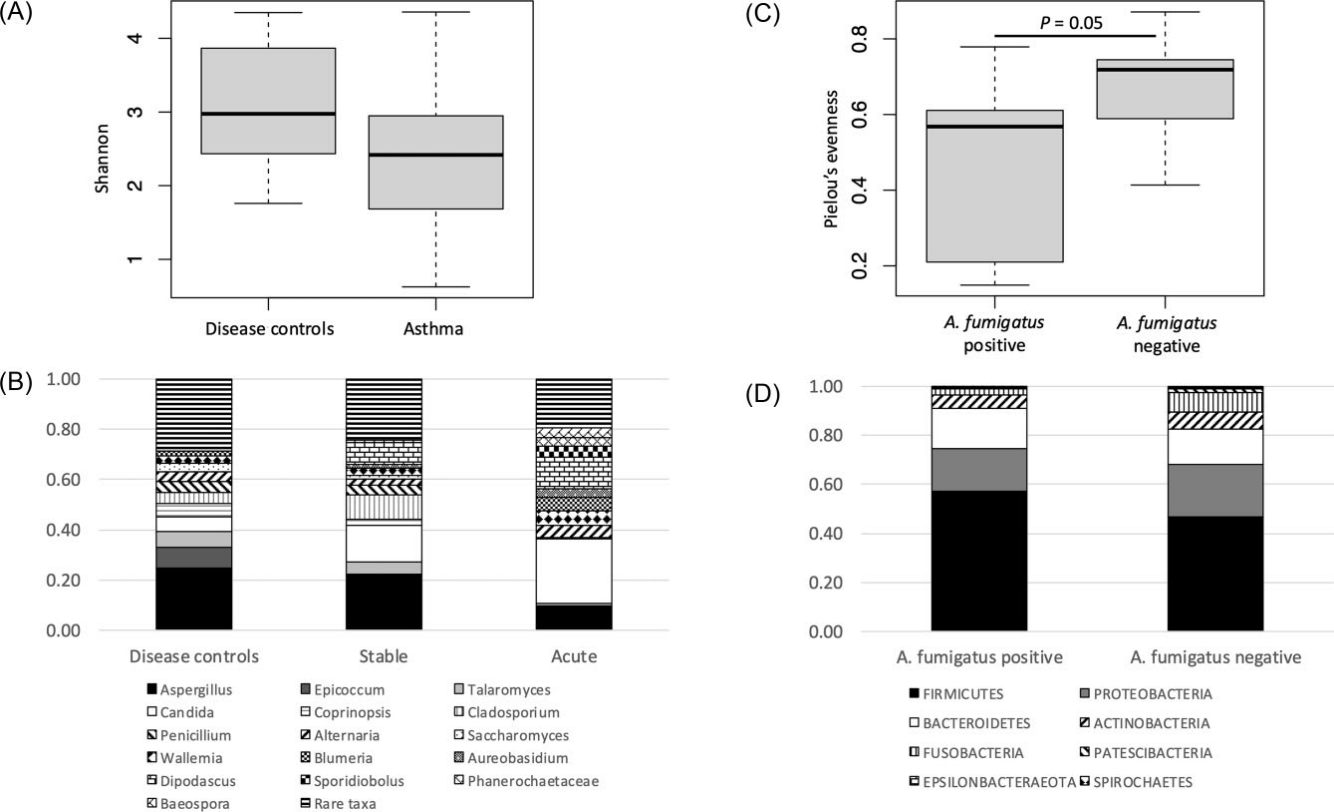
Relationship between airway fungal microbiota and clinical variables. (A) Boxplot of Shannon alpha diversity indices showing no significant difference in fungal diversity between asthma (*n* = 26) and disease controls (*n* = 8). (B) Stacked plot showing relative abundances of fungal genera in disease controls compared to stable and acute asthma. Genera with <4% mean relative abundance are classified as rare taxa. (C) Boxplot of Pielou’s evenness showing a significant difference in fungal alpha diversity between *A. fumigatus* culture positive and negative samples from sputum of asthmatics (*P* = .05). (D) Stacked plot showing relative abundances of bacterial phyla in the same asthmatic individuals in (C) with and without *A. fumigatus* culture.

There were no significant differences in alpha or beta diversity between asthma states or between asthma and disease controls, although there was a trend for lower alpha diversity in individuals with asthma (Fig. 2A). The stacked plot of genera in Figure 2B shows the increase in relative abundance of *Candida* from disease controls (6%) to stable (15%) through to acute asthma (26%), with a reduction in relative abundance of *Aspergillus* in stable and acute asthma compared to disease controls. Other notable differences between the three groups include the near absence of *Epicoccum* in asthma (8% in control compared to <0.5% in stable and acute asthma) and a reduction in rare taxa in asthma (genera <4% mean relative abundance in any asthma state) indicating a loss of diversity. Although there were no significant differences in fungal diversity between inhaled corticosteroids (ICS) doses, analysis by ANCOM found *A. fumigatus* to be differentially abundant between doses of ICS and disease controls (highest relative abundance in high [1000–2000 mg/day] and moderate [400–800 mg/day] ICS doses, respectively, and lowest in zero/low dose). With the asthma-only group, there was a significant difference in alpha diversity (*P* = .05) between *A. fumigatus* culture positive and negative samples, with the lowest diversity shown in the culture positive (Fig. 2C). A comparison of the bacterial phyla in the lungs of the same *A. fumigatus* culture-positive and culture-negative individuals showed that there was an increase in Firmicutes but a decrease in Fusobacteria in those culture positive for *A. fumigatus* compared to those who were culture negative (Fig. 2D).

### Effect of fungal sensitisation on airway bacterial and fungal microbiota

**Figure 3.**
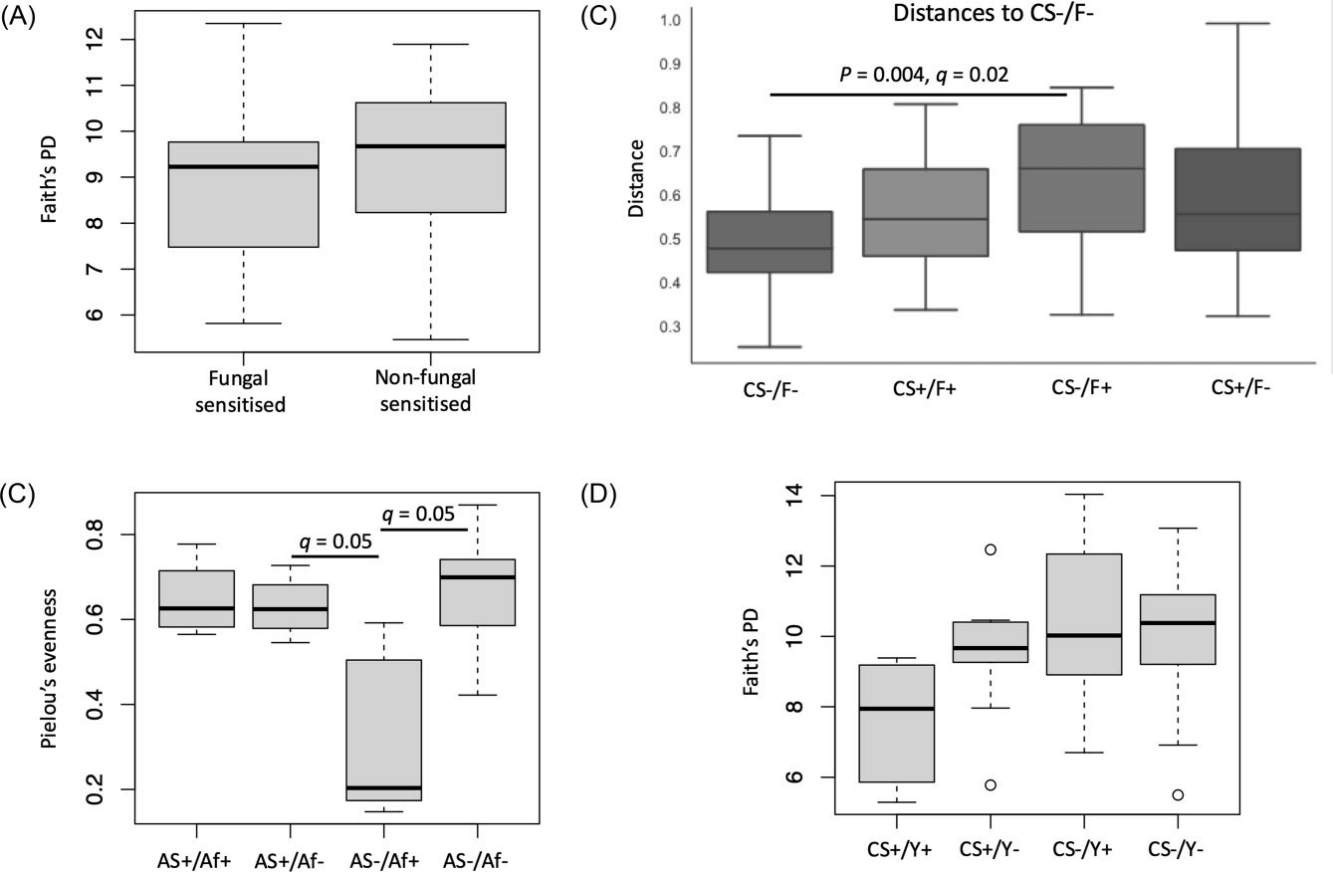
Effect of fungal sensitisation on bacterial and fungal microbiota in the lungs. (A) Boxplot of Faith’s phylogenetic diversity indices showing no significant difference in bacterial alpha diversity between fungal sensitised (*n* = 18) and non-fungal sensitised (*n* = 14) in the asthma-only group. (B) Boxplot of Pielou’s evenness indices showing a significantly lower fungal diversity in asthmatic individuals who were not sensitised to *A. fumigatus* with positive *A. fumigatus* culture than individuals with negative *A. fumigatus* culture who were either sensitised or not to *A. fumigatus* (*q* = 0.05). AS+, *A. fumigatus* sensitised; AS−, non-*A. fumigatus* sensitised; Af+, positive *A. fumigatus* culture from sputum; Af−, negative *A. fumigatus* culture from sputum. (C) Boxplots of bacterial Bray–Curtis dissimilarity showing the distance from non-*Candida* sensitised/culture negative for any fungus. A significant distance to non-*Candida* sensitised/culture positive for any fungus is shown (*P* = .004, *q* = 0.02). CS+, *Candida* sensitised; CS−, non-*Candida* sensitised; F+, positive culture of any fungi from sputum; F−, negative culture of any fungi from sputum. (D) Boxplot of Faith’s phylogenetic diversity indices showing no significant difference following multiple testing between *Candida* sensitised/yeast culture positive and all other groups (*P* = .02/.04, *q* = 0.08). CS+, *Candida* sensitised; CS−, non-*Candida* sensitised Y+, positive yeast culture; Y−, negative yeast culture.

Individuals who were fungal sensitised were categorised into those who were sensitised or not to *Candida* (Thermotolerant-fungal sensitised), *Penicillium* (Thermotolerant-filamentous fungal sensitised), *Cladosporium, Alternaria* (Filamentous fungal sensitised), and *A. fumigatus*. There was a significant difference in bacterial alpha diversity between *Candida* sensitised and non-*Candida* sensitised (*P* = .04) in the asthma and disease control group with the non-*Candida* sensitised having a higher diversity. Regarding fungal diversity, although there were no significant differences between fungal and non-fungal sensitised asthma, the proportion of individuals with >10% relative abundance of *Aspergillus* was greater in fungal sensitised (64%) than in non-fungal sensitised (33%) in the asthma-only group (Supplementary Fig. 1, S3). There were no significant differences in bacterial alpha diversity between fungal-sensitised and non-fungal-sensitised asthma (Fig. 3A).

The effects of yeast, *A. fumigatus* or the culture of any fungus from sputum on the microbial diversity of those sensitised or not to *Candida, A. fumigatus* or any fungi were investigated. No significant differences in diversity were observed with either bacterial or fungal microbiota in the asthma and disease control group. In the asthma-only group, however, fungal alpha diversity was significantly lower in samples with positive fungal culture of *A. fumigatus* from non-sensitised *A. fumigatus* (*q* = 0.05; Fig. 3B and Supplementary Fig. 2, S4) and non-sensitised *Candida* (*q* = 0.05) individuals than almost all other categories in their respective groupings. Additionally, ANCOM analysis identified *Candida albicans* and *Wallemia muriae* as differentially abundant in the *Candida*-sensitised/any fungal culture group.

With the bacterial microbiota there were significant beta diversity differences between individuals with positive and negative culture of any fungus of non-fungal sensitised (*P* = .007, *q* = 0.04), non-*Candida* sensitised (*P* = .004, *q* = 0.02; Fig. 3C), and non-*A. fumigatus* sensitised (*P* = .004, *q* = 0.02) in addition to significant beta diversity differences between positive and negative culture of yeast in non-*A. fumigatus* sensitised (*P* = .002, *q* = 0.01). *Candida*-sensitised individuals with positive yeast culture had a trend for lower bacterial alpha diversity than culture-negative *Candida* sensitised and all non-*Candida* sensitised, although this was not statistically significant after multiple testing correction (*P* = .02/.04, *q* = 0.08; Fig. 3D). Similarly, the effect of asthma severity and fungal sensitisation on microbial diversity was investigated by dividing asthma only and the asthma and disease control groups into fungal sensitised or not and GINA 0–3 or GINA 4–5 treatment groups. There was a trend for lower bacterial alpha diversity in fungal-sensitised individuals in GINA groups 4–5 than non-fungal sensitised in GINA groups 0–3, although this was not significant after multiple testing correction (*P* = .03, *q* = 0.17).

## Discussion

We characterised the lower airway bacterial and fungal microbiota of school-age asthmatics with and without fungal sensitisation along with paediatric disease controls. We found significant relationships between the airway microbiome and asthma severity, health status, and clinical features. Bacterial alpha diversity was significantly lower both in asthmatic individuals compared to disease controls and in acute asthma compared to stable asthma, showing a descending level of diversity from disease controls through stable to acute asthma. In addition, airway fungal culture positivity was associated with significant changes in microbial diversity.

To our knowledge, this is the first study to analyse the fungal and bacterial microbiota in the lungs of children with and without fungal sensitisation; however, we acknowledge the following limitations. Our study was based on relatively small numbers, which can increase the probability of a type-2 statistical error. Despite the relatively small sample size, a number of our findings are consistent with other airway microbiome studies. We found no significant changes in fungal diversity between disease controls and asthma states similar to previous studies in paediatric^20^ and adult asthma.^32^

In our study, *Aspergillus* and *Candida* were the most dominant of the airway mycobiota, and there was a notable increase in the relative abundance of *Candida* from disease controls to acute asthma, along with a decrease in *Aspergillus*, although no species from either genus were shown to be differentially abundant by ANCOM analysis. The higher relative abundance of *Aspergillus* in the disease controls compared to asthmatic patients is, however, at odds with our observed higher rates of *A. fumigatus* positive culture in asthma than controls. This could indicate that *Aspergillus* DNA amplified by PCR may not always be viable, and unlike positive fungal culture, it does not represent colonisation.^33^ Previous work on the lung mycobiome in asthma by Rick et al.^19^ also did not find any significant changes in *C. albicans* or *A. fumigatus* between asthma and health, whilst Sullivan et al.^34^ found that the level of *A. fumigatus* detected by qPCR in bronchoalveolar lavage samples was not related to asthma severity.

Culture of filamentous fungi from sputum (including *A. fumigatus*) has been associated with worse lung function in adult asthmatics,^7^ and so it was interesting to find that fungal diversity was significantly lower in asthmatic patients with positive *A. fumigatus* culture from sputum than those with no culture of *A. fumigatus*. In these same individuals, the bacterial phyla showed a shift in community structure from negative to positive culture of *A. fumigatus* with an increase in Firmicutes and a decrease in Fusobacteria that resembled the shifts in phyla from disease controls through to stable and acute asthma. It is known that *A. fumigatus* produces a variety of secondary metabolites that are thought to facilitate fungal growth by not only promoting IgE antibody-based inflammation by the ribotoxin Asp fI^35^ but also by inhibiting macrophage function through the action of mycotoxins such as gliotoxin.^36^ It may be that this immunosuppression could then lead to a less diverse fungal community as well as a shift in bacterial composition.

Our study did not observe any significant changes in microbial diversity or differentially abundant taxa between fungal-sensitised and non-fungal-sensitised asthmatic children similar to the findings of Rick et al.^19^ in adult asthmatics.

Since it has been reported that IgE sensitisation along with airway colonisation by *A. fumigatus* and *Candida* species can lead to AFAD,^5,33^ the effects of fungal culture from sputum on the microbial diversity of those sensitised or not to *A. fumigatus, Candida*, or any fungi were investigated. There were significant bacterial beta diversity differences between samples of positive and negative fungal culture from non-fungal, non-*Candida*, and non-*A. fumigatus*-sensitised asthmatics. Fungal alpha diversity was also found to be significantly lower in asthmatic individuals with positive cultures of *A. fumigatus* who were not sensitised to either *A. fumigatus* or *Candida*. It has already been discussed above how asthmatics with *A. fumigatus* positive culture have lower fungal diversity and a shift in bacterial phyla to an acute asthma profile that may be due to the secondary metabolites produced by *A. fumigatus* such as gliotoxin and the Asp fI ribotoxin, but this observation suggests that fungal sensitisation somehow maintains bacterial and fungal diversity during colonisation by *A. fumigatus*. Although the production of IgE anti-Asp fI antibodies in *A. fumigatus* sensitised asthmatics may augment Asp fI cytotoxic damage to respiratory mucosa,^35^ it may also prepare the immune response to *A. fumigatus* and inhibit colonisation of this fungus. In conclusion, we found that a positive airway fungal culture was associated with changes in the microbial diversity in children with asthma. Further studies should investigate whether treatment for *A. fumigatus* has a positive impact on the diversity of microbes in the lungs of children with asthma, especially those who are not fungal sensitised.

## Supplementary Material

myaf005_Supplemental_File
