# Supplementary material for: Positive sputum fungal culture, fungal sensitisation, and airway microbial diversity in asthmatic children
Source: Med Mycol. 2025 Jan 24;63(2):myaf005. doi: 10.1093/mmy/myaf005 (PMC11804241; doi:10.1093/mmy/myaf005)
Supplement: myaf005_Supplemental_File [file myaf005_supplemental_file.zip › mm-2024-0020-File005.docx]

**Supplemental Methods (S1)**

**Library preparation and sequencing**

Fungal ITS2

The ITS2 region of the nuclear ribosomal operon was amplified using barcoded TruGrade (Integrated DNA Technologies Inc., USA) ITS3 and ITS4 primers (Supplementary Table 1; White *et al* 1990) using a dual index nested PCR approach. Primer design incorporated a recognition sequence to allow for the nested PCR process. During the second round of PCR, Illumina adapter sequences and barcodes for sample identification based on the Illumina Nextera Index Adapter Sequences were incorporated (Supplementary Table 1). In the first round of PCR, each 20 µl reaction consisted of 10 µl KAPA HiFi HotStart Ready Mix (Kapa Biosystems, USA), 5 µl of template DNA and 0.25 µl of the barcoded ITS3 and ITS4 primers (10 µM each). Cycling conditions were 5 min at 95°C, followed by 18 cycles of 20 sec at 98°C, 20 sec at 62°C and 30 sec at 72°C, and then a final 5 min extension at 72°C. PCR reactions were set up in duplicate, and following pooling amplicons were purified using AxyPrep PCR Clean Up Beads (Axygen Biosciences, USA) in a 1:1 (beads:amplicon) ratio. Cleaned amplicons were eluted in 10 µl of PCR grade water and 9 µl used as the template for the second round of PCR, in a total volume of 20 µl including 10 µl KAPA HiFi HotStart Ready Mix and 0.5 µl of the forward and reverse barcoded Illumina adaptor incorporated primers (10 µM each). Cycling conditions were the same as round one, but with an annealing temperature of 65°C and 15 cycles. Products were cleaned with AxyPrep beads in a 1:1 ratio, quantified using the Qubit dsDNA HS assay kit (Thermo Fisher Scientific, UK) and the molarity of the region of interest calculated by running the products on a 2100 BioAnalyser using the High Sensitivity DNA kit (Agilent Technologies Inc., USA). The products were pooled in batches of up to 96 amplicons based on equal molar concentrations of the region of interest and submitted to the Centre for Genomic Research, University of Liverpool, for paired-end sequencing (2 x 300bp) on the Illumina MiSeq platform. Control samples included DNA extraction kit controls (PCR grade water subjected to DNA extraction protocol) and PCR reagent controls (PCR grade water treated as DNA for PCR amplification and cleaning steps). Sequence data, including those from controls were deposited in the European Nucleotide Archive (www.ebi.ac.uk/ena) under the study accession number PRJEB60938.

Bacterial 16S

Barcoded TruGrade (Integrated DNA Technologies Inc., USA) F515/R806 primers (Supplementary Table 1; Caporaso *et al* 2011) were used to amplify the V4 region of bacterial 16S ribosomal RNA using a similar dual index nested PCR approach as for ITS2. The cycling conditions for the primary PCR were: initial heating step at 95°C for 3 minutes, followed by 10 cycles of 98°C for 20 seconds, 65°C for 15 seconds and 72°C for 30 seconds. The final step included a 5-minute elongation phase at 72°C. Clean-up of amplicons and molarity calculation of the region of interest was performed as for ITS2 above. The nested secondary PCR cycling conditions were identical to the primary PCR except 15 cycles were used. The products were pooled in batches of up to 96 amplicons based on equal molar concentrations of the region of interest and submitted to the Centre for Genomic Research, University of Liverpool, for paired-end sequencing (2 x 250bp) on the Illumina MiSeq platform. Sequence data, including those from the controls, were deposited in the European Nucleotide Archive (www.ebi.ac.uk/ena) under the study accession number PRJEB60939.

**Bioinformatic and Statistical analyses**

Sequence processing incorporated recommended quality-filtering guidelines (Bokulich *et al* 2013). Primer sequences were removed using Cutadapt (version 1.9.1, Martin 2011). Sequences with a maximum primer error rate of 0.1 were retained and an overlap length of 8 bases between the read and the primer was accepted. Low-quality ends were removed from the reads using a quality cutoff of q30 before pairing R1 and R2 reads using a python script. BBMerge v38.51 (Bushnell *et al* 2017) was used to join the paired reads. Further processing using DADA2 (Callahan *et al* 2016) in QIIME2-2022.2 (Bolyen *et al* 2019) corrected sequencing errors, removed chimerics and singletons and dereplicated sequences to generate amplicon sequence variants (ASVs). Sequencing contaminants were identified from the negative PCR and kit controls and removed using the decontam package (default parameters) in R (Davis *et al*, 2018). Negative PCR and kit controls were removed before downstream analyses in QIIME2-2022.2. Pretrained Naïve Bayes classifiers were used to assign taxonomy using the SILVA database, release 132, 97% ASV for bacterial 16S (Quast *et al*, 2013) and QIIME release (version 8.2; 2020-02-20) of the UNITE database with dynamic clustering thresholds (Kõljalg *et al*, 2020; Nilsson *et al*, 2019) for fungal ITS2. ASVs without phyla level classification were removed.

To avoid sampling depth bias rarefied ASV tables were produced selecting the lowest number of reads within a sample for each group of samples. Samples were grouped together according to shared categorical variables. Alpha (within sample) and beta (between sample) diversity measurements were calculated for the rarefied ASV tables using the q2-diversity plugin. For 16S, Faith’s Phylogenetic Diversity and Pielou’s Evenness were used to measure alpha diversity and unweighted UniFrac distances were used to measure beta diversity. For ITS2, the non-phylogenetic Shannon’s diversity index in addition to Pielou’s Evenness were used for alpha diversity while for beta diversity the Bray-Curtis dissimilarity index was used. Significance testing for both 16S and ITS2 was performed using the non-parametric Kruskal-Wallis test for alpha diversity metrics and permutational multivariate analysis of variance (PERMANOVA) for beta diversity differences. Pairwise testing between all pairs of groups was performed with correction for multiple samples using the Benjamini-Hochberg FDR procedure generating q-values. Heatmaps of relative compositions were generated using the tidyverse package (Wickham *et al*, 2019) in R and stacked plots of relative compositions were created in Excel.

In order to identify taxa that were differentially abundant between sample groups, analysis of composition of microbiomes (ANCOM) was performed using unrarefied ASV tables (Mandal *et al*, 2015). For ANCOM the taxa frequency table was collapsed at genus level for 16S and species level for ITS and filtered to remove ASVs with less than 50 reads observed in less than 25% of samples in each comparison. Since ANCOM cannot tolerate ASV frequencies of zero, a pseudo-count composition table was created by adding a count of one to every value. The composition table was log-transformed and log-ratios calculated between pairwise combinations of taxa. ANCOM then summed how many times the null hypothesis (average abundance of a certain taxa not changing between groups) was rejected while controlling for false discoveries with a Benjamini-Hochberg correction at 5% level of significance. A volcano plot was produced for each comparison which relates the ‘W’ statistic (number of times null hypothesis rejected) to the clr (centre log ratio transformation) which represents the mean difference in abundance of a given taxon between sample groups.

**Supplementary Table 1.** Barcoded primer sequences used for ITS2 and 16S dual index nested PCR. Primary PCR primers incorporated a recognition sequence (underlined) to allow the secondary nested PCR. The secondary PCR incorporated the Illumina adapter sequences (italic) and an 8bp barcode (bold). Eight forward primers and twelve reverse primers created up to 96 different combinations.

| Primer name | Sequence |
| --- | --- |
| *Primary PCR* |  |
| ITS3forward | 5’ ACACTCTTTCCCTACACGACGCTCTTCCGATCTNNNNNGCATCGATGAAGAACGCAGC 3’ |
| ITS4reverse | 5’ GTGACTGGAGTTCAGACGTGTGCTCTTCCGATCTTCCTCCGCTTATTGATATGC 3’ |
| 16Sv4F1 | 5’ ACACTCTTTCCCTACACGACGCTCTTCCGATCTNNNNNGTGCCAGCMGCCGCGGTAA 3’ |
| 16Sv4R1 | 5’ GTGACTGGAGTTCAGACGTGTGCTCTTCCGATCTGGACTACHVGGGTWTCTAAT 3’ |
| *Secondary PCR* |  |
| DI_N501For | 5’ *AATGATACGGCGACCACCGAGATCTACAC***TAGATCGC**ACACTCTTTCCCTACACGACGCTC 3’ |
| DI_N502For | 5’ *AATGATACGGCGACCACCGAGATCTACAC***CTCTCTAT**ACACTCTTTCCCTACACGACGCTC 3’ |
| DI_N503For | 5’ *AATGATACGGCGACCACCGAGATCTACAC***TATCCTCT**ACACTCTTTCCCTACACGACGCTC 3’ |
| DI_N504For | 5’ *AATGATACGGCGACCACCGAGATCTACAC***AGAGTAGA**ACACTCTTTCCCTACACGACGCTC 3’ |
| DI_N505For | 5’ *AATGATACGGCGACCACCGAGATCTACAC***GTAAGGAG**ACACTCTTTCCCTACACGACGCTC 3’ |
| DI_N506For | 5’ *AATGATACGGCGACCACCGAGATCTACAC***ACTGCATA**ACACTCTTTCCCTACACGACGCTC 3’ |
| DI_N507For | 5’ *AATGATACGGCGACCACCGAGATCTACAC***AAGGAGTA**ACACTCTTTCCCTACACGACGCTC 3’ |
| DI_N508For | 5’ *AATGATACGGCGACCACCGAGATCTACAC***CTAAGCCT**ACACTCTTTCCCTACACGACGCTC 3’ |
| DI_N701Rev | 5’ *CAAGCAGAAGACGGCATACGAGAT***TCGCCTTA**GTGACTGGAGTTCAGACGTGTGCTC 3’ |
| DI_N702Rev | 5’ *CAAGCAGAAGACGGCATACGAGAT***CTAGTACG**GTGACTGGAGTTCAGACGTGTGCTC 3’ |
| DI_N703Rev | 5’ *CAAGCAGAAGACGGCATACGAGAT***TTCTGCCT**GTGACTGGAGTTCAGACGTGTGCTC 3’ |
| DI_N704Rev | 5’ *CAAGCAGAAGACGGCATACGAGAT***GCTCAGGA**GTGACTGGAGTTCAGACGTGTGCTC 3’ |
| DI_N705Rev | 5’ *CAAGCAGAAGACGGCATACGAGAT***AGGAGTCC**GTGACTGGAGTTCAGACGTGTGCTC 3’ |
| DI_N706Rev | 5’ *CAAGCAGAAGACGGCATACGAGAT***CATGCCTA**GTGACTGGAGTTCAGACGTGTGCTC 3’ |
| DI_N707Rev | 5’ *CAAGCAGAAGACGGCATACGAGAT***GTAGAGAG**GTGACTGGAGTTCAGACGTGTGCTC 3’ |
| DI_N708Rev | 5’ *CAAGCAGAAGACGGCATACGAGAT***CCTCTCTG**GTGACTGGAGTTCAGACGTGTGCTC 3’ |
| DI_N709Rev | 5’ *CAAGCAGAAGACGGCATACGAGAT***AGCGTAGC**GTGACTGGAGTTCAGACGTGTGCTC 3’ |
| DI_N710Rev | 5’ *CAAGCAGAAGACGGCATACGAGAT***CAGCCTCG**GTGACTGGAGTTCAGACGTGTGCTC 3’ |
| DI_N711Rev | 5’ *CAAGCAGAAGACGGCATACGAGAT***TGCCTCTT**GTGACTGGAGTTCAGACGTGTGCTC 3’ |
| DI_N712Rev | 5’ *CAAGCAGAAGACGGCATACGAGAT***TCCTCTAC**GTGACTGGAGTTCAGACGTGTGCTC 3’ |

**Supplemental References**

Bokulich NA, Subramanian S, Faith JJ*,* Gevers D, Gordon JI, Knight R et al. Quality-filtering vastly improves diversity estimates from Illumina amplicon sequencing*.* *Nat Methods* 2013; **10**: 57-59.

Bolyen E, Rideout JR, Dillon MR, Bokulich NA, Abnet CC, Al-Ghalith GA et al. Reproducible, interactive, scalable and extensible microbiome data science using QIIME 2. *Nat Biotech* 2019; **37:** 852–857.

Bushnell B, Rood J, Singer E. BBMerge–accurate paired shotgun read merging via overlap. *PloS ONE* 2017; **12**.

Callahan BJ, McMurdie PJ, Rosen MJ, Han AW, Johnson AJA, Holmes SP. DADA2: High-resolution sample inference from Illumina amplicon data. Nat Methods 2016; **13**: 581-583.

Caporaso JG, Lauber CL, Walters WA, Berg-Lyons D, Lozupone CA, Turnbaugh PJ et al. Global patterns of 16S rRNA diversity at a depth of millions of sequences per sample. *Proc Natl Acad Sci U S A* 2011; **108**: 4516-4522.

Davis NM, Proctor DM, Holmes SP, Relman DA, Callahan BJ. Simple statistical identification and removal of contaminant sequences in marker-gene and metagenomics data. *Microbiome* 2018; **6:** 226.

Kõljalg U, Nilsson HR, Schigel D, Tedersoo L, Larsson KH, May TW et al. The Taxon Hypothesis Paradigm—On the Unambiguous Detection and Communication of Taxa. *Microorganisms* 2020; **8:** 1910.

Mandal S, Van Treuren W, White RA*,* Eggesbø M, Knight R, Peddada SD. Analysis of composition of microbiomes: a novel method for studying microbial composition*.* *Microb Ecol Health Dis* 2015; **26**: 27663.

Martin M. Cutadapt removes adapter sequences from high-throughput sequencing reads*.* *EMBnet journal* 2011; **17**: 10-12.

Nilsson RH, Larsson K-H, Taylor AFS, Bengtsson-Palme J, Jeppesen TS, Schigel D et al. The UNITE database for molecular identification of fungi: handling dark taxa and parallel taxonomic classifications. *Nucleic Acids Research* 2019; **47:** D259-D264.

Quast C, Pruesse E, Yilmaz P, Gerken J, Schweer T, Yarza P, *et al*. The SILVA ribosomal RNA gene database project: improved data processing and web-based tools. *Nucleic Acids Research* 2013; **41:** D590-D596.

White TJ, Bruns T, Lee S, Taylor J. Amplification and direct sequencing of fungal ribosomal RNA genes for phylogenetics. In: Innis MA, Gelfand DH, Sninsky JJ, White TJ eds. *PCR protocols: A guide to Methods and Applications.* New York: Academic Press, 1990: 315-322.

Wickham H, Averick M, Bryan J, Chang W, McGowen LA, François R et al. Welcome to the Tidyverse. *The Journal of Open Source Software* 2019; **4:** 1686.
