# Supplementary material for: Positive sputum fungal culture, fungal sensitisation, and airway microbial diversity in asthmatic children
Source: Med Mycol. 2025 Jan 24;63(2):myaf005. doi: 10.1093/mmy/myaf005 (PMC11804241; doi:10.1093/mmy/myaf005)
Supplement: myaf005_Supplemental_File [file myaf005_supplemental_file.zip › mm-2024-0020-File006.docx]

**Supplementary Tables (S2)**

**Supplementary Table 2. ANCOM and alpha and beta diversity analysis of bacterial microbiota from samples grouped together according to shared categorical variables (Asthma and healthy control group).**

|  |  | **n** | **Alpha diversity (Faith’s)** | | **Alpha diversity (Evenness)** | | **Beta diversity** | | **ANCOM**  **identified taxa** | **W value** | **Clr**  **value** |
| --- | --- | --- | --- | --- | --- | --- | --- | --- | --- | --- | --- |
|  |  |  | **All groups *P*-value** | **Pairwise *q*-value** | **All groups *P*-value** | **Pairwise *q*-value** | **All groups *P*-value** | **Pairwise *q*-value** |  |  |  |
| Asthma | Yes | 34 | **0.02** | - | 0.98 | - | 0.74 | - | - | - | - |
|  | No | 15 |  |  |  |  |  |  |  |  |  |
| Subject group | Healthy (0) | 15 | **0.02** | **0.03 (0&2)** | 0.24 | - | 0.62 | - | *Corynebacterium* | 18 | 8.5 |
|  | Stable asthma (1) | 27 |  |  |  |  |  |  | *Delftia* | 9 | 7.6 |
|  | Acute asthma (2) | 7 |  |  |  |  |  |  | *Lactobacillus* | 4 | 6.5 |
|  |  |  |  |  |  |  |  |  | *Mogibacterium* | 4 | 4.4 |
| Age | 5-8 (1) | 11 | 0.31 | - | **0.008** | **0.004 (2&3)** | 0.05 | **0.01 (2&3)** | *Streptobacillus* | 23 | 11.8 |
|  | 9-12 (2) | 20 |  |  |  |  |  |  | *Olsenella* | 4 | 2.9 |
|  | 13-16 (3) | 18 |  |  |  |  |  |  |  |  |  |
| Sex | Female | 23 | 0.06 | - | 0.55 | - | 0.41 | - | - | - | - |
|  | Male | 26 |  |  |  |  |  |  |  |  |  |
| GINA group | 0 (healthy) | 15 | **0.04** | **0.01 (0&4-5)** | 0.93 | - | 0.79 | - | *Moraxella* | 4 | 6.5 |
|  | 1-3 (stable) | 20 |  |  |  |  |  |  |  |  |  |
|  | 4-5 (severe) | 14 |  |  |  |  |  |  |  |  |  |
| Mould in home | Yes | 28 | 0.39 | **-** | 0.86 | - | 0.91 | - | - | - | - |
|  | No | 16 |  |  |  |  |  |  |  |  |  |
| ICS dose (mg/day) | Healthy (0) | 15 | 0.07 | **0.05 (0&3)** | 0.33 | - | 0.86 | - | - | - | - |
|  | 0-200 (1) | 7 |  |  |  |  |  |  |  |  |  |
|  | 400-800 (2) | 16 |  |  |  |  |  |  |  |  |  |
|  | 1000-2000 (3) | 11 |  |  |  |  |  |  |  |  |  |
| Any culture | Yes | 26 | 0.64 | - | 0.53 | - | 0.13 | - | - | - | - |
|  | No | 23 |  |  |  |  |  |  |  |  |  |
| Yeast culture | Yes | 17 | 0.22 | - | 0.95 | - | 0.46 | - | *Pseudomonas* | 11 | 1.5 |
|  | No | 32 |  |  |  |  |  |  |  |  |  |
| Af culture | Yes | 11 | 0.63 | - | 0.29 | - | 0.96 | - | - | - | - |
|  | No | 38 |  |  |  |  |  |  |  |  |  |
| Non-Af fil culture | Yes | 3 | 0.93 | - | **0.03** | - | 0.31 | - | - | - | - |
|  | No | 46 |  |  |  |  |  |  |  |  |  |
| Any fil culture | Yes | 13 | 0.54 | - | 0.84 | - | 0.81 | - | - | - | - |
|  | No | 36 |  |  |  |  |  |  |  |  |  |
| Atopic | Yes | 30 | **0.004** | - | 0.58 | - | 0.4 | - | - | - | - |
|  | No | 18 |  |  |  |  |  |  |  |  |  |
| Atopy | None (0) | 16 | **0.01** | **0.009 (0&2)** | 0.69 | - | 0.71 | - | - | - | - |
|  | NF allergen (1) | 13 |  |  |  |  |  |  |  |  |  |
|  | NF&F allergen (2) | 17 |  |  |  |  |  |  |  |  |  |
| Fungal sensitised | Yes | 18 | **0.04** | - | 0.26 | - | 0.73 | - | - | - | - |
|  | No | 29 |  |  |  |  |  |  |  |  |  |
| Filamentous FS | Yes | 16 | 0.2 | - | 0.13 | - | 0.65 | - | - | - | - |
|  | No | 31 |  |  |  |  |  |  |  |  |  |
| Thermo fil FS | Yes | 11 | 0.43 | - | 0.37 | - | 0.64 | - | - | - | - |
|  | No | 36 |  |  |  |  |  |  |  |  |  |
| Thermo FS | Yes | 14 | **0.04** | - | 0.31 | - | 0.55 | - | - | - | - |
|  | No | 33 |  |  |  |  |  |  |  |  |  |
| Af sensitised | Yes | 10 | 0.56 | - | 0.68 | - | 0.63 | - | - | - | - |
|  | No | 37 |  |  |  |  |  |  |  |  |  |
| Fungal sensitised/GINA | Sensitised/GINA 0-3 (1) | 9 | 0.07 | - | 0.64 | - | 0.85 | - | - | - | - |
|  | Sensitised/GINA 4-5 (2) | 9 |  |  |  |  |  |  |  |  |  |
|  | Non-sensitised/GINA 0-3 (3) | 25 |  |  |  |  |  |  |  |  |  |
|  | Non-sensitised/GINA 0-3 (4) | 4 |  |  |  |  |  |  |  |  |  |
| Fungal sensitised/ any culture | Sensitised/Positive culture (1) | 10 | 0.24 | - | 0.51 | - | 0.34 | - | - | - | - |
|  | Sensitised/Negative culture (2) | 8 |  |  |  |  |  |  |  |  |  |
|  | Non-sensitised/Positive culture (3) | 14 |  |  |  |  |  |  |  |  |  |
|  | Non-sensitised/Negative culture (4) | 15 |  |  |  |  |  |  |  |  |  |
| Fungal sensitised/ *Af* culture | Sensitised/Positive culture (1) | 7 | 0.21 | - | 0.35 | - | 0.76 | - | - | - |  |
|  | Sensitised/Negative culture (2) | 11 |  |  |  |  |  |  |  |  |  |
|  | Non-sensitised/Positive culture (3) | 4 |  |  |  |  |  |  |  |  |  |
|  | Non-sensitised/Negative culture (4) | 25 |  |  |  |  |  |  |  |  |  |
| Fungal sensitised/ yeast culture | Sensitised/Positive culture (1) | 5 | 0.13 | - | 0.49 | - | 0.56 | - | - | - | - |
|  | Sensitised/Negative culture (2) | 13 |  |  |  |  |  |  |  |  |  |
|  | Non-sensitised/Positive culture (3) | 10 |  |  |  |  |  |  |  |  |  |
|  | Non-sensitised/Negative culture (4) | 19 |  |  |  |  |  |  |  |  |  |
| *Candida* sensitised/any culture | Sensitised/Positive culture (1) | 9 | 0.21 | - | 0.61 | - | 0.07 | - | - | - | - |
|  | Sensitised/Negative culture (2) | 5 |  |  |  |  |  |  |  |  |  |
|  | Non-sensitised/Positive culture (3) | 15 |  |  |  |  |  |  |  |  |  |
|  | Non-sensitised/Negative culture (4) | 18 |  |  |  |  |  |  |  |  |  |
| *Candida* sensitised/*Af* culture | Sensitised/Positive culture (1) | 6 | 0.3 | - | 0.67 | - | 0.65 | - | - | - | - |
|  | Sensitised/Negative culture (2) | 8 |  |  |  |  |  |  |  |  |  |
|  | Non-sensitised/Positive culture (3) | 5 |  |  |  |  |  |  |  |  |  |
|  | Non-sensitised/Negative culture (4) | 28 |  |  |  |  |  |  |  |  |  |
| *Candida* sensitised/yeast culture | Sensitised/Positive culture (1) | 5 | 0.07 | - | 0.68 | - | 0.46 | - | - | - | - |
|  | Sensitised/Negative culture (2) | 9 |  |  |  |  |  |  |  |  |  |
|  | Non-sensitised/Positive culture (3) | 10 |  |  |  |  |  |  |  |  |  |
|  | Non-sensitised/Negative culture (4) | 23 |  |  |  |  |  |  |  |  |  |
| *Af* sensitised/any culture | Sensitised/Positive culture (1) | 6 | 0.98 | - | 0.86 | - | 0.08 | - | - | - | - |
|  | Sensitised/Negative culture (2) | 4 |  |  |  |  |  |  |  |  |  |
|  | Non-sensitised/Positive culture (3) | 18 |  |  |  |  |  |  |  |  |  |
|  | Non-sensitised/Negative culture (4) | 19 |  |  |  |  |  |  |  |  |  |
| *Af* sensitised/*Af* culture | Sensitised/Positive culture (1) | 4 | 0.76 | - | 0.77 | - | 0.73 | - | - | - | - |
|  | Sensitised/Negative culture (2) | 6 |  |  |  |  |  |  |  |  |  |
|  | Non-sensitised/Positive culture (3) | 7 |  |  |  |  |  |  |  |  |  |
|  | Non-sensitised/Negative culture (4) | 30 |  |  |  |  |  |  |  |  |  |
| *Af* sensitised/yeast culture | Sensitised/Positive culture (1) | 3 | 0.71 | - | 0.94 | - | 0.56 | - | - | - | - |
|  | Sensitised/Negative culture (2) | 7 |  |  |  |  |  |  |  |  |  |
|  | Non-sensitised/Positive culture (3) | 12 |  |  |  |  |  |  |  |  |  |
|  | Non-sensitised/Negative culture (4) | 25 |  |  |  |  |  |  |  |  |  |

Significance testing was performed using the non-parametric Kruskal-Wallis test for alpha diversity metrics and permutational multivariate analysis of variance (PERMANOVA) for beta diversity differences. Pairwise testing between all pairs of groups was performed with correction for multiple samples using the Benjamini-Hochberg FDR procedure generating q-values. Significant *P*-values (<0.05) are shown in bold. Bacteria differentially abundant between categorical variables were identified by analysis of composition of microbiomes (ANCOM) analysis. The W value represents the number of times the null hypothesis (no change in abundance between groups) has been rejected, whilst the clr (centre log ratio transformed) value represents the mean difference in abundance of a certain taxon between variables.

**Supplementary Table 3. ANCOM and alpha and beta diversity analysis of bacterial microbiota from samples grouped together according to shared categorical variables (Asthma only group).**

| **Category** | **Variables** | **n** | **Alpha diversity (Faith’s)** | | **Alpha diversity (Evenness)** | | **Beta diversity** | | **ANCOM**  **identified taxa** | **W value** | **Clr**  **value** |
| --- | --- | --- | --- | --- | --- | --- | --- | --- | --- | --- | --- |
|  |  |  | **All groups *P*-value** | **Pairwise *q*-value** | **All groups *P*-value** | **Pairwise *q*-value** | **All groups *P*-value** | **Pairwise *q*-value** |  |  |  |
| Subject group | Stable asthma | 7 | **0.03** | **-** | 0.12 | - | 0.47 | - | *Corynebacterium* | 14 | 2.2 |
|  | Acute asthma | 27 |  |  |  |  |  |  | *Solobacterium* | 7 | 1.7 |
|  |  |  |  |  |  |  |  |  | *Delftia* | 5 | 1.6 |
|  |  |  |  |  |  |  |  |  | *Clostridiales* | 4 | 2.1 |
|  |  |  |  |  |  |  |  |  | *Haemophilus* | 3 | 1.3 |
| Age | 5-8 (1) | 10 | 0.33 | - | **0.05** | **0.01 (2&3)** | 0.18 | - | *Streptobacillus* | 6 | 10.2 |
|  | 9-12 (2) | 14 |  |  |  |  |  |  | Ruminococcaceae | 2 | 5.3 |
|  | 13-16 (3) | 10 |  |  |  |  |  |  | *Parvimonas* | 2 | 4 |
| Sex | Female | 13 | 0.87 | - | 0.68 | - | 0.87 | - | - | - | - |
|  | Male | 21 |  |  |  |  |  |  |  |  |  |
| GINA group | 1-3 (stable) | 20 | 0.29 | **-** | 0.65 | - | 0.72 | - | Candidate div SR1 | 3 | 1.8 |
|  | 4-5 (severe) | 14 |  |  |  |  |  |  |  |  |  |
| Mould in home | Yes | 17 | 0.09 | - | 0.91 | - | 0.99 | - | - | - | - |
|  | No | 11 |  |  |  |  |  |  |  |  |  |
| ICS dose (mg/day) | 0-200 (1) | 7 | 0.46 | - | 0.15 | - | 0.83 | - | - | - | - |
|  | 400-800 (2) | 16 |  |  |  |  |  |  |  |  |  |
|  | 1000-2000 (3) | 11 |  |  |  |  |  |  |  |  |  |
| Any culture | Yes | 17 | 0.81 | - | 0.89 | - | 0.15 | - | - | - | - |
|  | No | 15 |  |  |  |  |  |  |  |  |  |
| Yeast culture | Yes | 13 | 0.13 | - | 0.71 | - | 0.58 | - | - | - | - |
|  | No | 21 |  |  |  |  |  |  |  |  |  |
| Af culture | Yes | 9 | 0.86 | - | 0.2 | - | 0.95 | - | - | - | - |
|  | No | 25 |  |  |  |  |  |  |  |  |  |
| Non-Af fil culture | Yes | 2 | 0.83 | - | 0.19 | - | 0.58 | - | - | - | - |
|  | No | 32 |  |  |  |  |  |  |  |  |  |
| Any fil culture | Yes | 10 | 0.82 | - | 0.29 | - | 0.73 | - | - | - | - |
|  | No | 24 |  |  |  |  |  |  |  |  |  |
| Atopic | Yes | 25 | 0.17 | - | 0.36 | - | 0.98 | - | - | - | - |
|  | No | 8 |  |  |  |  |  |  |  |  |  |
| Atopy | None (0) | 6 | 0.45 | - | 0.66 | - | 0.7 | - | - | - | - |
|  | NF allergen (1) | 8 |  |  |  |  |  |  |  |  |  |
|  | NF&F allergen (2) | 17 |  |  |  |  |  |  |  |  |  |
| Fungal sensitised | Yes | 18 | 0.22 | - | 0.31 | - | 0.66 | - | - | - | - |
|  | No | 14 |  |  |  |  |  |  |  |  |  |
| Filamentous FS | Yes | 16 | 0.62 | - | 0.19 | - | 0.71 | - | - | - | - |
|  | No | 16 |  |  |  |  |  |  |  |  |  |
| Thermo fil FS | Yes | 11 | 0.67 | - | 0.49 | - | 0.79 | - | - | - | - |
|  | No | 21 |  |  |  |  |  |  |  |  |  |
| Thermo FS | Yes | 14 | 0.11 | - | 0.34 | - | 0.54 | - | - | - | - |
|  | No | 18 |  |  |  |  |  |  |  |  |  |
| *Af* sensitised | Yes | 10 | 0.94 | - | 0.81 | - | 0.79 | - | - | - | - |
|  | No | 37 |  |  |  |  |  |  |  |  |  |
| Fungal sensitised/GINA | Sensitised/GINA 0-3 (1) | 9 | 0.48 | - | 0.55 | - | 0.83 | **-** | - | - | - |
|  | Sensitised/GINA 4-5 (2) | 9 |  |  |  |  |  |  |  |  |  |
|  | Non-sensitised/GINA 0-3 (3) | 10 |  |  |  |  |  |  |  |  |  |
|  | Non-sensitised/GINA 0-3 (4) | 4 |  |  |  |  |  |  |  |  |  |
| Fungal sensitised/ any culture | Sensitised/Positive culture (1) | 10 | 0.77 | - | 0.45 | - | 0.19 | **0.04 (3&4)** | - | - | - |
|  | Sensitised/Negative culture (2) | 8 |  |  |  |  |  |  |  |  |  |
|  | Non-sensitised/Positive culture (3) | 7 |  |  |  |  |  |  |  |  |  |
|  | Non-sensitised/Positive culture (4) | 7 |  |  |  |  |  |  |  |  |  |
| Fungal sensitised/ *Af* culture | Sensitised/Positive culture (1) | 7 | 0.7 | - | 0.35 | - | 0.93 | - | - | - | - |
|  | Sensitised/Negative culture (2) | 11 |  |  |  |  |  |  |  |  |  |
|  | Non-sensitised/Positive culture (3) | 2 |  |  |  |  |  |  |  |  |  |
|  | Non-sensitised/Positive culture (4) | 12 |  |  |  |  |  |  |  |  |  |
| Fungal sensitised/ yeast culture | Sensitised/Positive culture (1) | 5 | 0.35 | - | 0.53 | - | 0.26 | - | - | - | - |
|  | Sensitised/Negative culture (2) | 13 |  |  |  |  |  |  |  |  |  |
|  | Non-sensitised/Positive culture (3) | 6 |  |  |  |  |  |  |  |  |  |
|  | Non-sensitised/Negative culture (4) | 8 |  |  |  |  |  |  |  |  |  |
| *Candida* sensitised/any culture | Sensitised/Positive culture (1) | 9 | 0.61 | - | 0.76 | - | **0.04** | **0.02 (3&4)** | - | - | - |
|  | Sensitised/Negative culture (2) | 5 |  |  |  |  |  |  |  |  |  |
|  | Non-sensitised/Positive culture (3) | 8 |  |  |  |  |  |  |  |  |  |
|  | Non-sensitised/Negative culture (4) | 10 |  |  |  |  |  |  |  |  |  |
| *Candida* sensitised/*Af* culture | Sensitised/Positive culture (1) | 6 | 0.42 | - | 0.58 | - | 0.87 | - | - | - | - |
|  | Sensitised/Negative culture (2) | 8 |  |  |  |  |  |  |  |  |  |
|  | Non-sensitised/Positive culture (3) | 3 |  |  |  |  |  |  |  |  |  |
|  | Non-sensitised/Negative culture (4) | 15 |  |  |  |  |  |  |  |  |  |
| *Candida* sensitised/yeast culture | Sensitised/Positive culture (1) | 5 | 0.45 | - | 0.61 | - | 0.36 | - | - | - | - |
|  | Sensitised/Negative culture (2) | 9 |  |  |  |  |  |  |  |  |  |
|  | Non-sensitised/Positive culture (3) | 6 |  |  |  |  |  |  |  |  |  |
|  | Non-sensitised/Negative culture (4) | 12 |  |  |  |  |  |  |  |  |  |
| *Af* sensitised/any culture | Sensitised/Positive culture (1) | 6 | 0.99 | - | 0.89 | - | 0.07 | **0.02 (3&4)** | - | - | - |
|  | Sensitised/Negative culture (2) | 4 |  |  |  |  |  |  |  |  |  |
|  | Non-sensitised/Positive culture (3) | 11 |  |  |  |  |  |  |  |  |  |
|  | Non-sensitised/Negative culture (4) | 11 |  |  |  |  |  |  |  |  |  |
| *Af* sensitised/*Af* culture | Sensitised/Positive culture (1) | 4 | 0.44 | - | 0.72 | - | 0.98 | - | - | - | - |
|  | Sensitised/Negative culture (2) | 6 |  |  |  |  |  |  |  |  |  |
|  | Non-sensitised/Positive culture (3) | 5 |  |  |  |  |  |  |  |  |  |
|  | Non-sensitised/Negative culture (4) | 17 |  |  |  |  |  |  |  |  |  |
| *Af* sensitised/yeast culture | Sensitised/Positive culture (1) | 4 | 1 | - | 0.95 | - | 0.09 | **0.01 (3&4)** | - | - | - |
|  | Sensitised/Negative culture (2) | 6 |  |  |  |  |  |  |  |  |  |
|  | Non-sensitised/Positive culture (3) | 8 |  |  |  |  |  |  |  |  |  |
|  | Non-sensitised/Negative culture (4) | 14 |  |  |  |  |  |  |  |  |  |

Significance testing was performed using the non-parametric Kruskal-Wallis test for alpha diversity metrics and permutational multivariate analysis of variance (PERMANOVA) for beta diversity differences. Pairwise testing between all pairs of groups was performed with correction for multiple samples using the Benjamini-Hochberg FDR procedure generating q-values. Significant *P*-values (<0.05) are shown in bold. Bacteria differentially abundant between categorical variables were identified by analysis of composition of microbiomes (ANCOM) analysis. The W value represents the number of times the null hypothesis (no change in abundance between groups) has been rejected, whilst the clr (centre log ratio transformed) value represents the mean difference in abundance of a certain taxon between variables.

**Supplementary Table 4. ANCOM and alpha and beta diversity analysis of fungal microbiota from samples grouped together according to shared categorical variables (Asthma and healthy control group).**

|  |  | **n** | **Alpha diversity (Faith’s)** | | **Alpha diversity (Evenness)** | | **Beta diversity** | | **ANCOM**  **identified taxa** | **W value** | **Clr**  **value** |
| --- | --- | --- | --- | --- | --- | --- | --- | --- | --- | --- | --- |
|  |  |  | **All groups *P*-value** | **Pairwise *q*-value** | **All groups *P*-value** | **Pairwise *q*-value** | **All groups *P*-value** | **Pairwise *q*-value** |  |  |  |
| Asthma | Yes | 26 | 0.27 | - | 0.24 | - | 0.48 | - | - | - | - |
|  | No | 8 |  |  |  |  |  |  |  |  |  |
| Subject group | Healthy (0) | 8 | 0.55 | **-** | 0.5 | - | 0.87 | - | *-* | - | - |
|  | Stable asthma (1) | 6 |  |  |  |  |  |  |  |  |  |
|  | Acute asthma (2) | 20 |  |  |  |  |  |  |  |  |  |
| Age | 5-8 (1) | 8 | 0.89 | - | 0.67 | - | 0.11 | - | *-* | - | - |
|  | 9-12 (2) | 12 |  |  |  |  |  |  |  |  |  |
|  | 13-16 (3) | 14 |  |  |  |  |  |  |  |  |  |
| Sex | Female | 15 | **0.05** | - | 0.44 | - | 0.36 | - | *-* | - | - |
|  | Male | 19 |  |  |  |  |  |  |  |  |  |
| GINA group | 0 (healthy) | 8 | 0.48 | - | 0.48 | - | 0.52 | - | *-* | - | - |
|  | 1-3 (stable) | 14 |  |  |  |  |  |  |  |  |  |
|  | 4-5 (severe) | 12 |  |  |  |  |  |  |  |  |  |
| Mould in home | Yes | 18 | 0.28 | - | 0.68 | - | 0.22 | - | *-* | - | - |
|  | No | 13 |  |  |  |  |  |  |  |  |  |
| ICS dose (mg/day) | Healthy (0) | 8 | 0.45 | - | 0.69 | - | 0.92 | - | *Aspergillus fumigatus* |  |  |
|  | 0-200 (1) | 5 |  |  |  |  |  |  | *A. terreus* |  |  |
|  | 400-800 (2) | 12 |  |  |  |  |  |  |  |  |  |
|  | 1000-2000 (3) | 9 |  |  |  |  |  |  |  |  |  |
| Any culture | Yes | 20 | 0.75 | - | 0.22 | - | 0.27 | - | *-* | - | - |
|  | No | 14 |  |  |  |  |  |  |  |  |  |
| Yeast culture | Yes | 13 | 0.28 | - | 0.68 | - | 0.22 | - | *-* | - | - |
|  | No | 21 |  |  |  |  |  |  |  |  |  |
| Af culture | Yes | 10 | 0.17 | - | 0.08 | - | 0.51 | - | *-* | - | - |
|  | No | 24 |  |  |  |  |  |  |  |  |  |
| Non-Af fil culture | Yes | 2 | 0.51 | - | 0.46 | - | 0.46 | - | *-* | - | - |
|  | No | 32 |  |  |  |  |  |  |  |  |  |
| Any fil culture | Yes | 10 | 0.17 | - | 0.08 | - | 0.51 | - | *-* | - | - |
|  | No | 24 |  |  |  |  |  |  |  |  |  |
| Atopic | Yes | 25 | 0.68 | - | 0.86 | - | 0.22 | - | *-* | - | - |
|  | No | 9 |  |  |  |  |  |  |  |  |  |
| Atopy | None (0) | 8 | 0.98 | - | 0.97 | - | 0.61 | - | *-* | - | - |
|  | NF allergen (1) | 12 |  |  |  |  |  |  |  |  |  |
|  | NF&F allergen (2) | 13 |  |  |  |  |  |  |  |  |  |
| Fungal sensitised | Yes | 14 | 0.65 | - | 0.89 | - | 0.76 | - | *-* | - | - |
|  | No | 20 |  |  |  |  |  |  |  |  |  |
| Filamentous FS | Yes | 16 | 0.69 | - | 0.94 | - | 0.85 | - | *-* | - | - |
|  | No | 31 |  |  |  |  |  |  |  |  |  |
| Thermo fil FS | Yes | 11 | 0.52 | - | 0.98 | - | 0.72 | - | *-* | - | - |
|  | No | 36 |  |  |  |  |  |  |  |  |  |
| Thermo FS | Yes | 14 | 0.72 | - | 0.86 | - | 0.66 | - | *-* | - | - |
|  | No | 33 |  |  |  |  |  |  |  |  |  |
| Af sensitised | Yes | 10 | 0.54 | - | 0.8 | - | 0.44 | - | *-* | - | - |
|  | No | 37 |  |  |  |  |  |  |  |  |  |
| Fungal sensitised/GINA | Sensitised/GINA 0-3 (1) | 6 | 0.8 | - | 0.83 | - | 0.71 | - | - | - | - |
|  | Sensitised/GINA 4-5 (2) | 8 |  |  |  |  |  |  |  |  |  |
|  | Non-sensitised/GINA 0-3 (3) | 16 |  |  |  |  |  |  |  |  |  |
|  | Non-sensitised/GINA 0-3 (4) | 4 |  |  |  |  |  |  |  |  |  |
| Fungal sensitised/ any culture | Sensitised/Positive culture (1) | 10 | 0.75 | - | 0.56 | - | 0.79 | - | - | - | - |
|  | Sensitised/Negative culture (2) | 4 |  |  |  |  |  |  |  |  |  |
|  | Non-sensitised/Positive culture (3) | 10 |  |  |  |  |  |  |  |  |  |
|  | Non-sensitised/Positive culture (4) | 10 |  |  |  |  |  |  |  |  |  |
| Fungal sensitised/ *Af* culture | Sensitised/Positive culture (1) | 7 | 0.54 | - | 0.5 | - | 0.91 | - | - | - | - |
|  | Sensitised/Negative culture (2) | 7 |  |  |  |  |  |  |  |  |  |
|  | Non-sensitised/Positive culture (3) | 3 |  |  |  |  |  |  |  |  |  |
|  | Non-sensitised/Positive culture (4) | 17 |  |  |  |  |  |  |  |  |  |
| Fungal sensitised/ yeast culture | Sensitised/Positive culture (1) | 5 | 0.63 | - | 0.89 | - | 0.67 | - | - | - | - |
|  | Sensitised/Negative culture (2) | 9 |  |  |  |  |  |  |  |  |  |
|  | Non-sensitised/Positive culture (3) | 8 |  |  |  |  |  |  |  |  |  |
|  | Non-sensitised/Negative culture (4) | 12 |  |  |  |  |  |  |  |  |  |
| *Candida* sensitised/any culture | Sensitised/Positive culture (1) | 9 | 0.93 | - | 0.71 | - | 0.45 | - | - | - | - |
|  | Sensitised/Negative culture (2) | 3 |  |  |  |  |  |  |  |  |  |
|  | Non-sensitised/Positive culture (3) | 11 |  |  |  |  |  |  |  |  |  |
|  | Non-sensitised/Negative culture (4) | 11 |  |  |  |  |  |  |  |  |  |
| *Candida* sensitised/*Af* culture | Sensitised/Positive culture (1) | 6 | 0.3 | - | 0.28 | - | 0.71 | - | - | - | - |
|  | Sensitised/Negative culture (2) | 6 |  |  |  |  |  |  |  |  |  |
|  | Non-sensitised/Positive culture (3) | 4 |  |  |  |  |  |  |  |  |  |
|  | Non-sensitised/Negative culture (4) | 18 |  |  |  |  |  |  |  |  |  |
| *Candida* sensitised/yeast culture | Sensitised/Positive culture (1) | 5 | 0.66 | - | 0.95 | - | 0.75 | - | - | - | - |
|  | Sensitised/Negative culture (2) | 7 |  |  |  |  |  |  |  |  |  |
|  | Non-sensitised/Positive culture (3) | 8 |  |  |  |  |  |  |  |  |  |
|  | Non-sensitised/Negative culture (4) | 14 |  |  |  |  |  |  |  |  |  |
| *Af* sensitised/any culture | Sensitised/Positive culture (1) | 6 | 0.93 | - | 0.62 | - | 0.13 | - | - | - | - |
|  | Sensitised/Negative culture (2) | 2 |  |  |  |  |  |  |  |  |  |
|  | Non-sensitised/Positive culture (3) | 14 |  |  |  |  |  |  |  |  |  |
|  | Non-sensitised/Negative culture (4) | 12 |  |  |  |  |  |  |  |  |  |
| *Af* sensitised/*Af* culture | Sensitised/Positive culture (1) | 4 | 0.24 | - | 0.19 | - | 0.46 | - | - | - | - |
|  | Sensitised/Negative culture (2) | 4 |  |  |  |  |  |  |  |  |  |
|  | Non-sensitised/Positive culture (3) | 6 |  |  |  |  |  |  |  |  |  |
|  | Non-sensitised/Negative culture (4) | 20 |  |  |  |  |  |  |  |  |  |
| *Af* sensitised/yeast culture | Sensitised/Positive culture (1) | 3 | 0.26 | - | 0.8 | - | 0.38 | - | - | - | - |
|  | Sensitised/Negative culture (2) | 5 |  |  |  |  |  |  |  |  |  |
|  | Non-sensitised/Positive culture (3) | 10 |  |  |  |  |  |  |  |  |  |
|  | Non-sensitised/Negative culture (4) | 16 |  |  |  |  |  |  |  |  |  |

Significance testing was performed using the non-parametric Kruskal-Wallis test for alpha diversity metrics and permutational multivariate analysis of variance (PERMANOVA) for beta diversity differences. Pairwise testing between all pairs of groups was performed with correction for multiple samples using the Benjamini-Hochberg FDR procedure generating q-values. Significant *P*-values (<0.05) are shown in bold. Fungi differentially abundant between categorical variables were identified by analysis of composition of microbiomes (ANCOM) analysis. The W value represents the number of times the null hypothesis (no change in abundance between groups) has been rejected, whilst the clr (centre log ratio transformed) value represents the mean difference in abundance of a certain taxon between variables.

**Supplementary Table 5. ANCOM and alpha and beta diversity analysis of fungal microbiota from samples grouped together according to shared categorical variables (Asthma only group).**

| **Category** | **Variables** | **n** | **Alpha diversity (Faith’s)** | | **Alpha diversity (Evenness)** | | **Beta diversity** | | **ANCOM**  **identified taxa** | **W value** | **Clr**  **value** |
| --- | --- | --- | --- | --- | --- | --- | --- | --- | --- | --- | --- |
|  |  |  | **All groups *P*-value** | **Pairwise *q*-value** | **All groups *P*-value** | **Pairwise *q*-value** | **All groups *P*-value** | **Pairwise *q*-value** |  |  |  |
| Subject group | Stable asthma | 6 | 0.95 | - | 0.9 | - | 0.92 | - | *-* | - | - |
|  | Acute asthma | 20 |  |  |  |  |  |  |  |  |  |
| Age | 5-8 (1) | 7 | 0.7 | - | 1 | - | 0.22 | - | *-* | - | - |
|  | 9-12 (2) | 8 |  |  |  |  |  |  |  |  |  |
|  | 13-16 (3) | 11 |  |  |  |  |  |  |  |  |  |
| Sex | Female | 10 | **0.02** | - | 0.11 | - | 0.08 | - | *Dipodascus geotrichum* | 2 | 4.5 |
|  | Male | 16 |  |  |  |  |  |  |  |  |  |
| GINA group | 1-3 (stable) | 14 | 0.54 | **-** | 0.88 | - | 0.48 | - | *-* | - | - |
|  | 4-5 (severe) | 12 |  |  |  |  |  |  |  |  |  |
| Mould in home | Yes | 12 | 0.21 | - | 0.39 | - | 0.88 | - | *-* | - | - |
|  | No | 11 |  |  |  |  |  |  |  |  |  |
| ICS dose (mg/day) | 0-200 (1) | 5 | 0.39 | - | 0.65 | - | 0.93 | - | *A. fumigatus* | 1 | 4.2 |
|  | 400-800 (2) | 12 |  |  |  |  |  |  | *A. terreus* | 1 | 1.8 |
|  | 1000-2000 (3) | 9 |  |  |  |  |  |  |  |  |  |
| Any culture | Yes | 17 | 0.57 | - | 0.17 | - | 0.44 | - | *-* | - | - |
|  | No | 9 |  |  |  |  |  |  |  |  |  |
| Yeast culture | Yes | 11 | 0.48 | - | 0.7 | - | 0.21 | - | *Candida albicans* | 1 | 4.1 |
|  | No | 15 |  |  |  |  |  |  | *Mycospharella tassiana* | 1 | 1.8 |
| Af culture | Yes | 9 | 0.08 | - | **0.05** | - | 0.53 | - | - | - | - |
|  | No | 17 |  |  |  |  |  |  |  |  |  |
| Non-Af fil culture | Yes | 2 | 0.56 | - | 0.77 | - | 0.46 | - | - | - | - |
|  | No | 24 |  |  |  |  |  |  |  |  |  |
| Any fil culture | Yes | 9 | 0.08 | - | **0.05** | - | 0.56 | - | - | - | - |
|  | No | 17 |  |  |  |  |  |  |  |  |  |
| Atopic | Yes | 22 | **0.01** | - | **0.01** | - | 0.4 | - | - | - | - |
|  | No | 4 |  |  |  |  |  |  |  |  |  |
| Atopy | None (0) | 3 | 0.1 | 0.07 (0&1/0&2) | 0.11 | 0.08 (0&1/0&2) | 0.53 | - | - | - | - |
|  | NF allergen (1) | 9 |  |  |  |  |  |  |  |  |  |
|  | NF&F allergen (2) | 13 |  |  |  |  |  |  |  |  |  |
| Fungal sensitised | Yes | 14 | 0.88 | - | 0.57 | - | 0.95 | - | - | - | - |
|  | No | 12 |  |  |  |  |  |  |  |  |  |
| Filamentous FS | Yes | 12 | 0.92 | - | 0.54 | - | 0.89 | - | - | - | - |
|  | No | 14 |  |  |  |  |  |  |  |  |  |
| Thermo fil FS | Yes | 9 | 0.73 | - | 0.5 | - | 0.75 | - | - | - | - |
|  | No | 17 |  |  |  |  |  |  |  |  |  |
| Thermo FS | Yes | 12 | 0.88 | - | 0.64 | - | 0.88 | - | - | - | - |
|  | No | 14 |  |  |  |  |  |  |  |  |  |
| *Af* sensitised | Yes | 8 | 0.74 | - | 0.66 | - | 0.58 | - | - | - | - |
|  | No | 18 |  |  |  |  |  |  |  |  |  |
| Fungal sensitised/GINA | Sensitised/GINA 0-3 (1) | 6 | 0.79 | - | 0.93 | - | 0.83 | - | - | - | - |
|  | Sensitised/GINA 4-5 (2) | 8 |  |  |  |  |  |  |  |  |  |
|  | Non-sensitised/GINA 0-3 (3) | 8 |  |  |  |  |  |  |  |  |  |
|  | Non-sensitised/GINA 0-3 (4) | 4 |  |  |  |  |  |  |  |  |  |
| Fungal sensitised/ any culture | Sensitised/Positive culture (1) | 10 | 0.95 | - | 0.5 | - | 0.9 | - | - | - | - |
|  | Sensitised/Negative culture (2) | 4 |  |  |  |  |  |  |  |  |  |
|  | Non-sensitised/Positive culture (3) | 7 |  |  |  |  |  |  |  |  |  |
|  | Non-sensitised/ Negative culture (4) | 5 |  |  |  |  |  |  |  |  |  |
| Fungal sensitised/ *Af* culture | Sensitised/Positive culture (1) | 7 | 0.11 | - | 0.09 | - | 0.94 | - | - | - | - |
|  | Sensitised/Negative culture (2) | 7 |  |  |  |  |  |  |  |  |  |
|  | Non-sensitised/Positive culture (3) | 2 |  |  |  |  |  |  |  |  |  |
|  | Non-sensitised/ Negative culture (4) | 10 |  |  |  |  |  |  |  |  |  |
| Fungal sensitised/ yeast culture | Sensitised/Positive culture (1) | 5 | 0.63 | - | 0.89 | - | 0.63 | - | - | - | - |
|  | Sensitised/Negative culture (2) | 9 |  |  |  |  |  |  |  |  |  |
|  | Non-sensitised/Positive culture (3) | 8 |  |  |  |  |  |  |  |  |  |
|  | Non-sensitised/ Negative culture (4) | 12 |  |  |  |  |  |  |  |  |  |
| *Candida* sensitised/ any culture | Sensitised/Positive culture (1) | 9 | 0.84 | - | 0.4 | - | 0.6 | - | - | - | - |
|  | Sensitised/Negative culture (2) | 3 |  |  |  |  |  |  |  |  |  |
|  | Non-sensitised/Positive culture (3) | 8 |  |  |  |  |  |  |  |  |  |
|  | Non-sensitised/ Negative culture (4) | 6 |  |  |  |  |  |  |  |  |  |
| *Candida* sensitised/ *Af* culture | Sensitised/Positive culture (1) | 6 | **0.04** | **0.04 (1&3)**  **0.04 (3&4)** | **0.04** | **0.04 (1&3)**  **0.04 (2&3)**  **0.04 (3&4)** | 0.79 | - | - | - | - |
|  | Sensitised/Negative culture (2) | 6 |  |  |  |  |  |  |  |  |  |
|  | Non-sensitised/Positive culture (3) | 3 |  |  |  |  |  |  |  |  |  |
|  | Non-sensitised/ Negative culture (4) | 11 |  |  |  |  |  |  |  |  |  |
| *Candida* sensitised/ yeast culture | Sensitised/Positive culture (1) | 5 | 0.91 | - | 0.89 | - | 0.84 | - | - | - | - |
|  | Sensitised/Negative culture (2) | 7 |  |  |  |  |  |  |  |  |  |
|  | Non-sensitised/Positive culture (3) | 6 |  |  |  |  |  |  |  |  |  |
|  | Non-sensitised/ Negative culture (4) | 8 |  |  |  |  |  |  |  |  |  |
| *Af* sensitised/ any culture | Sensitised/Positive culture (1) | 6 | 0.96 | - | 0.46 | - | 0.12 | - | - | - | - |
|  | Sensitised/Negative culture (2) | 2 |  |  |  |  |  |  |  |  |  |
|  | Non-sensitised/Positive culture (3) | 11 |  |  |  |  |  |  |  |  |  |
|  | Non-sensitised/ Negative culture (4) | 7 |  |  |  |  |  |  |  |  |  |
| *Af* sensitised/ *Af* culture | Sensitised/Positive culture (1) | 4 | 0.1 | - | **0.03** | **0.05 (2&3)**  **0.05 (3&4)** | 0.46 | - | - | - | - |
|  | Sensitised/Negative culture (2) | 4 |  |  |  |  |  |  |  |  |  |
|  | Non-sensitised/Positive culture (3) | 5 |  |  |  |  |  |  |  |  |  |
|  | Non-sensitised/ Negative culture (4) | 13 |  |  |  |  |  |  |  |  |  |
| *Af* sensitised/ yeast culture | Sensitised/Positive culture (1) | 3 | 0.32 | - | 0.71 | - | 0.36 | - | - | - | - |
|  | Sensitised/Negative culture (2) | 5 |  |  |  |  |  |  |  |  |  |
|  | Non-sensitised/Positive culture (3) | 8 |  |  |  |  |  |  |  |  |  |
|  | Non-sensitised/ Negative culture (4) | 10 |  |  |  |  |  |  |  |  |  |

Significance testing was performed using the non-parametric Kruskal-Wallis test for alpha diversity metrics and permutational multivariate analysis of variance (PERMANOVA) for beta diversity differences. Pairwise testing between all pairs of groups was performed with correction for multiple samples using the Benjamini-Hochberg FDR procedure generating q-values. Significant *P*-values (<0.05) are shown in bold. Fungi differentially abundant between categorical variables were identified by analysis of composition of microbiomes (ANCOM) analysis. The W value represents the number of times the null hypothesis (no change in abundance between groups) has been rejected, whilst the clr (centre log ratio transformed) value represents the mean difference in abundance of a certain taxon between variables.
