# Supplementary material for: Positive sputum fungal culture, fungal sensitisation, and airway microbial diversity in asthmatic children
Source: Med Mycol. 2025 Jan 24;63(2):myaf005. doi: 10.1093/mmy/myaf005 (PMC11804241; doi:10.1093/mmy/myaf005)
Supplement: myaf005_Supplemental_File [file myaf005_supplemental_file.zip › mm-2024-0020-File007.docx]

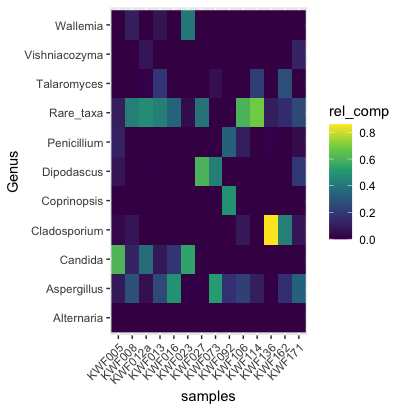

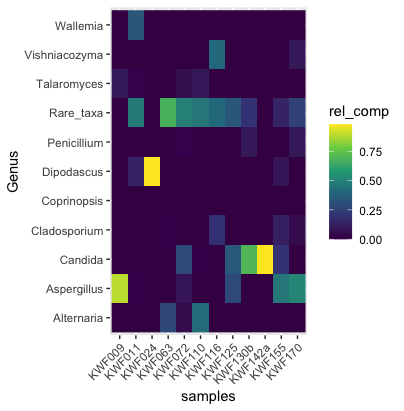


Fungal sensitised

Non-fungal sensitised

**Supplementary Figure 1.** Heatmap showing relative abundances of fungal genera in fungal sensitised and non-fungal sensitised asthmatic children. Genera with <4% relative abundance in any of the groups are classified as rare taxa.
