# Supplementary material for: Positive sputum fungal culture, fungal sensitisation, and airway microbial diversity in asthmatic children
Source: Med Mycol. 2025 Jan 24;63(2):myaf005. doi: 10.1093/mmy/myaf005 (PMC11804241; doi:10.1093/mmy/myaf005)
Supplement: myaf005_Supplemental_File [file myaf005_supplemental_file.zip › mm-2024-0020-File008.docx]

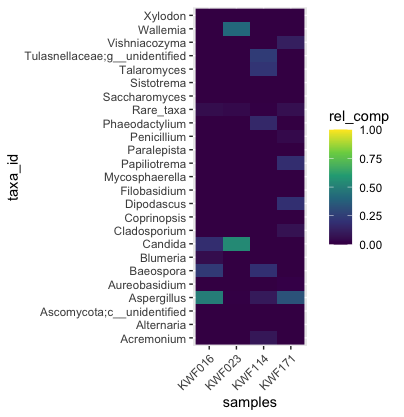

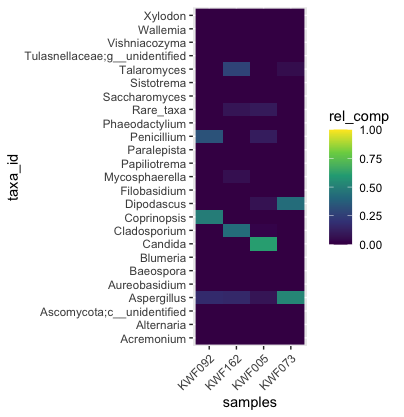

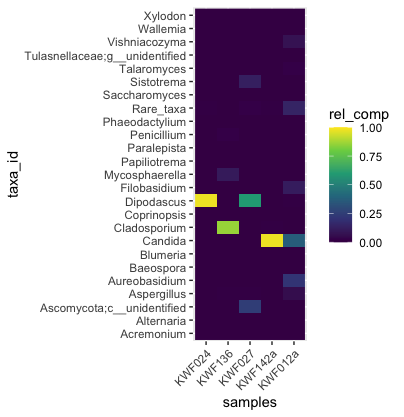

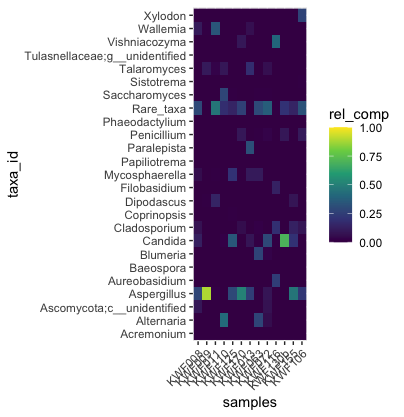


AFS+/Af+

AFS+/Af-

AFS-/Af+

AFS-/Af-

**Supplementary Figure 2.** Heatmaps showing relative abundances of fungal genera in asthmatic individuals sensitised or not to *Aspergillus fumigatus* with and without positive *A. fumigatus* culture from sputum. AFS+, *A. fumigatus* sensitised; AFS-, non-*A. fumigatus* sensitised; Af+, positive fungal culture from sputum; Af-, negative fungal culture from sputum
